# Microstructural Development in a Laser-Remelted Al-Zn-Si-Mg Coating

**DOI:** 10.1038/s41598-017-15847-y

**Published:** 2017-11-23

**Authors:** M. Godec, B. Podgornik, D. Nolan

**Affiliations:** 1 Institute of Metals and Technology, Lepi pot 11, 1000 Ljubljana, Slovenia; 2 Bluescope Ltd, PO Box 1854, Wollongong, NSW 2500 Australia

## Abstract

In the last five decades, there has been intense development in the field of Zn-Al galvanic coating modification. Recently, Mg was added to improve corrosion properties. Further improvements to the coating are possible with additional laser surface treatment. In this article, we focus on remelting the Al-Zn-Mg-Si layer, using a diode laser with a wide-beam format, concentrating on the microstructure development during extreme cooling rates. Laser remelting of the Al-Zn-Mg-Si coating and rapid self-quenching produces a finer grain size, and a microstructure that is substantially refined and homogenized with respect to the phase distribution. Using EBSD results, we are able to understand microstructure modification. The laser modified coating has some porosity and intergranular cracking which are difficult to avoid, however this does not seem to be detrimental to mechanical properties, such as ductility on bending. The newly developed technology has a high potential for improved corrosion performance due to highly refined microstructure.

## Introduction

The Zn-55%Al-1.6%Si coating for sheet steel was commercialized in the 1970s as an improvement over the traditional Zn-based coating. Since that time, its improved corrosion resistance has led to widespread use, such that the total production capacity exceeded 135 million metric tonnes in 2014^1^. The success of this coating system is due to its unique combination of physico-chemical properties: the barrier protection effect of an interfacial intermetallic layer at the coating/steel interface, an aluminium-rich dendritic matrix, and the sacrificial properties of a Zn-rich interdendritic component^2,3^.

The intermetallic layer forms first as the steel strip is submerged into the galvanizing bath. It is based on Al-Fe-Si phases and continues to grow during solidification^4^. The solidification process of the coating overlay is initiated by the formation of aluminium nuclei on the intermetallic layer and the growth of the primary phase through the coating thickness and then laterally in two dimensions, parallel to the steel sheet^5^. It has been shown that there is no texture correlation between the substrate and the coating overlay, and the crystallographic misorientation within a single grain is relatively high, especially in the large grains^5,6^.

The corrosion resistance of this coating can be improved by an increased cooling rate during solidification, which results in a refinement of the coating microstructure^7^ and more effective retention of the corrosion products in the interdendritic regions during service. However, in a practical sense, the cooling rate achievable in a high-speed manufacturing environment is typically less than 100 °C, under which conditions the dendrite-arm spacing will be approximately 7 µm^8^. Using a high-power diode laser, it has proven possible to rapidly remelt and self-quench the coating without affecting the steel substrate, thereby achieving cooling rates of the order of 10^5^ °C/s and producing refined microstructures with a dendrite-arm spacing of the order of 1 µm^9,10^. This has been shown to result in improved corrosion performance with both bare and painted products, a benefit that has been somewhat counteracted by the unpredictable appearance of porosity, and a moderate detrimental effect on the coating’s ductility due to the rapid quenching^11^.

In 2013, the BlueScope Steel Ltd commercialised an improved formulation of the Al-Zn coating with the composition Zn-55%Al-2%Mg-1.6%Si. The addition of Mg led to significant improvements in the corrosion performance across a broad range of applications and environments, in both the bare and painted product conditions^12^. One of the key mechanisms for a performance improvement is the formation of new Mg-containing phases in the interdendritic regions of the coating microstructure, such as Mg_2_Si and MgZn_2_. These phases act to enhance the corrosion resistance in a number of ways. For example, the Mg released from the dissolution of the highly active MgZn_2_ phase during corrosion forms stable corrosion products on cathodic sites, thereby reducing the driving force for further corrosion. Secondly, the stable Mg_2_Si phase forms in the interdenritic channels in the lower part of the coatings, providing a barrier to continued corrosion of the Zn-rich phases from these interdendritic paths. Also, the presence of Mg during solidification causes a refinement of the coating’s microstructure, leading to a moderate reduction in the secondary dendrite-arm spacing. The effect of these mechanisms in combination is to improve the corrosion performance through a reduction in the overall corrosion rate. This novel alloying strategy has made it possible to reduce the coating mass for both bare and pre-painted products, while maintaining improvements in the corrosion performance.

To further improve the corrosion performance of the Mg-containing Al-Zn coating, researchers have recently applied the findings of previous work on laser refinement to the new coating formulation. Some limited evaluation of the microstructure has been reported that proves the dendrite-arm spacing is indeed further refined by the laser-remelting process. It was also shown that a more complex mix of phases results, and the implications for this on the corrosion performance are yet to be determined^13^.

The current work aims to extend the limited literature relating to the laser refinement of Al-Zn-Si-Mg coatings, especially in terms of grain growth and texture development. The aim of the current work was to characterize the microstructure of an Al-Zn-Mg-Si coating remelted using a diode laser with a wide-beam format, focusing on the microstructure development during extreme cooling rates.

## Experimental Details

### Material and Laser Remelting

**Figure 1:**
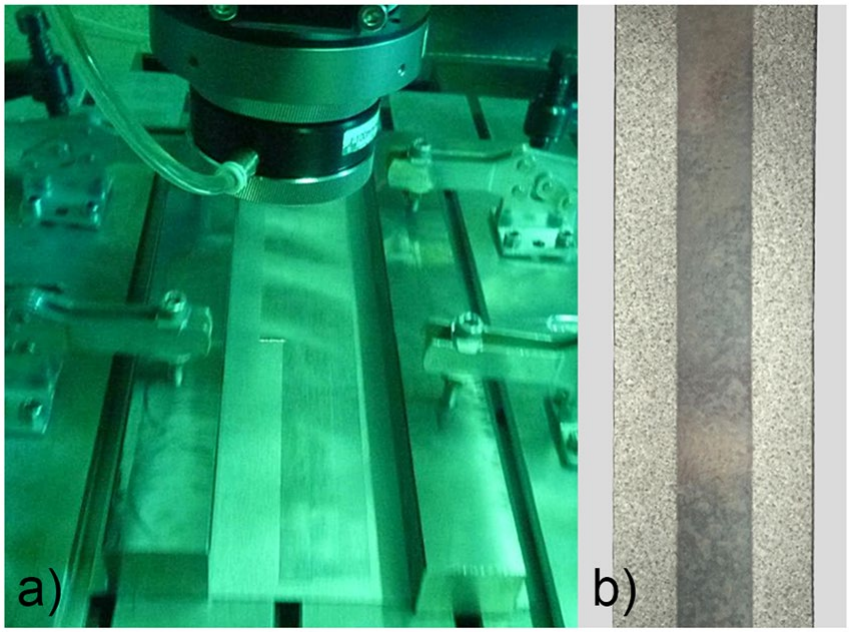
(**a**) Laser-remelting setup and (**b**) Al-Zn-Si-Mg hot-dip galvanized sample with laser-remelted area.

A commercial hot-dip galvanizing process was used to produce a Zn-55%Al-2%Mg-1.5%Si coating on a 0.42 mm thick steel sheet. The nominal coating mass was 75 g/m^2^, on each side, which equates to an approximately 20-µm-thick coating. A 10-kW, high-power diode laser from Laserline GmbH was used to remelt the coating, using a power level of 5 kW and a wide beam with spot size of 11.0 × 0.6 mm. The travel speed was 9 m/min and heat input was 3 J/mm^2^. Each coated steel sheet sample for laser treatment was approximately 400 mm long and 150 mm wide. Informed by the previous work of Mathew *et al*.^9^, we assumed that because complete melting of coating was achieved, and overheating to the extent of causing intermetallic layer growth or recrystallization of the cold-formed steel substrate was avoided, then the peak temperature of the coating layer during processing was between 560 °C and 650 °C. Figure 1(a) shows the experimental setup for the remelting process, with the laser-remelted area in the middle of the sample clearly visible in Fig. 1(b). The laser treatment was conducted in air. Both as-received and laser-remelted coatings were analysed in terms of the microstructure and texture analysis.

### Microstructure Analysis

The samples for the SEM studies of the surfaces were analysed in the as-received state, only cleaning the surface with alcohol in an ultrasound bath. Samples for cross-sectional analyses were prepared either by mechanical polishing to 1 µm diamond paste, or ion etched using a PECS Gatan 682 device. The surface preparation procedure for the top-view EBSD analysis used to analyse the grain size and the grain growth was as follows. The as-received samples were ion etched at 6 kV for 3 minutes, followed by ion etching at 2 kV for 20 minutes. For the cross-section EBSD analysis focused on the microstructural defects, mechanical grinding and polishing was followed by a final ion polishing using a Jeol cross-section polisher.

Microstructural, EDS and EBSD analyses were performed using a FEG SEM JEOL 6500 F field-emission scanning electron microscope with energy-dispersive spectroscopy (an INCA X-SIGHT LH2-type detector, INCA ENERGY 450 software) and EBSD (HKL Nordlys II EBSD camera using Channel5 software). The instrument was operated at 15 kV and a 1.2-nA current for the EBSD analysis, with a tilting angle of 70 degrees. The aluminium IPF phase orientation map was observed using cross-section and top view. The detection was set to 5–7 bands, 4 × 4 binning. Minimal post-processing was performed in the case of the mappings, which was limited to removing the so-called “wild spikes”. The laser-remelted structure was also characterised in a JEOL 2011FX TEM at 200 kV. TEM samples, 100 nm thick, were prepared using an xT Nova NanoLab 200 Dualbeam focused-ion-beam miller at 30 kV.

The coating’s ductility on bending was evaluated using the 2 T test, where the bend radius is equivalent to the thickness of the steel substrate (0.42 mm). SE images of the bend surfaces were produced for a comparison of the crack severity.

## Results and Discussions

### Microstructure

**Figure 2:**
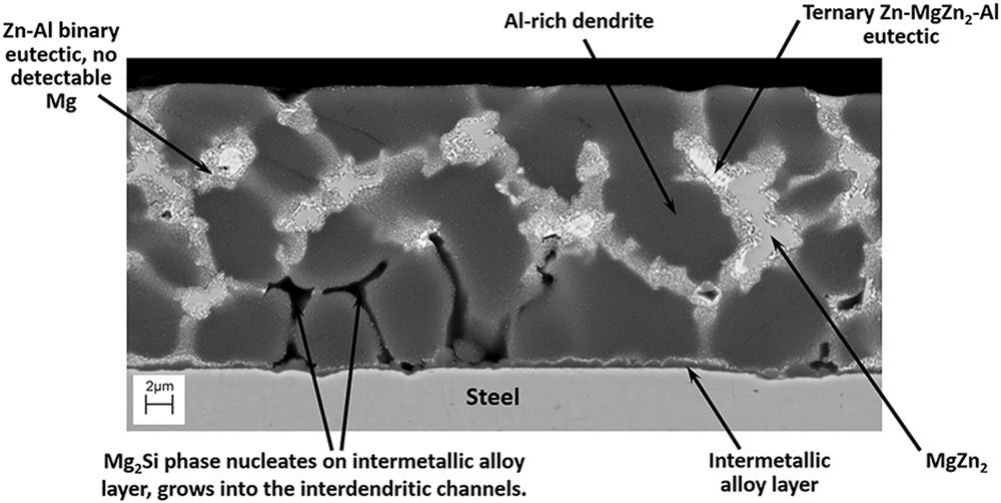
Microstructure of Al-Zn-Mg-Si continuous hot-dip coating. (After Nolan^14^).

The microstructure of the Al-Zn-Mg-Si continuous hot-dip coating is complex. The structure of the as-received coating in the cross-section is shown in Fig. 2. A thin intermetallic layer is formed on the surface of the steel sheet during immersion in the galvanizing bath, and during the initial stages of nucleation. This is followed by heterogeneous nucleation of Al-rich dendrites (primary phase) on the intermetallic layer. The Mg_2_Si phase nucleates on the intermetallic alloy layer and grows into the interdendritic channels. During solidification, and due to the non-equilibrium segregation effects, the liquid composition shifts towards the Zn-Al binary eutectic, which then forms at the interface between the aluminium-rich dendrites and the remaining liquid. Finally, the remaining liquid phase in the interdendritic channels becomes enriched in Zn and Mg, to the extent that the MgZn_2_ phase is formed in a complex, divorced ternary eutectic with Zn and Al.

Al-Zn-Mg-Si coatings form a typical grain (spangle) topography, resulting from the formation of primary dendrites. The spangle size of the coatings depends on the constitution of the galvanizing bath during the hot-dip processing. Mg additions of 2% are known to have a moderate effect on reducing the spangle size.

**Figure 3:**
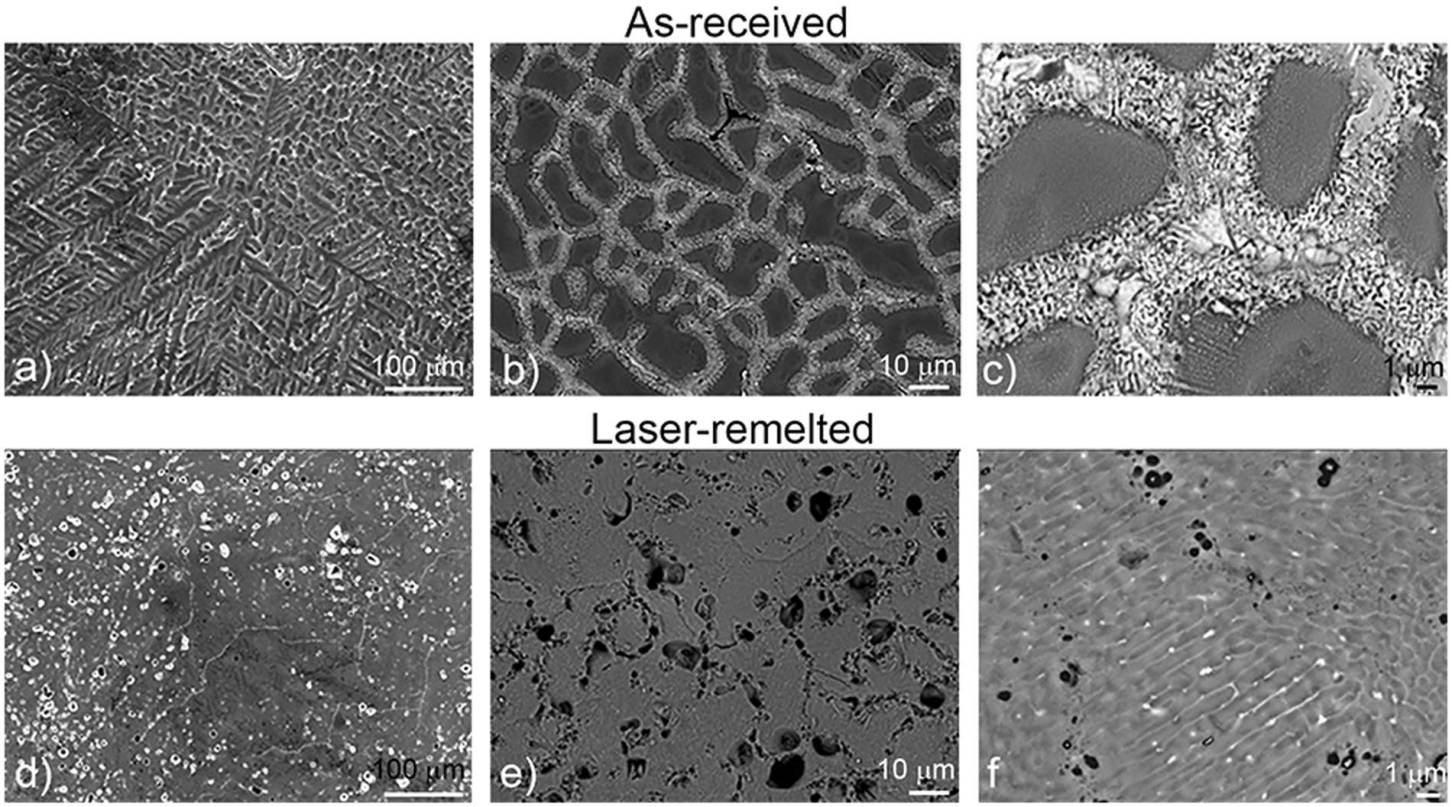
Top view SE and BE images obtained by SEM of as-received (**a** is SE image, **b** and **c** are BE images) and laser-remelted (**d** is SE, **e** and **f** are BE images) coating.

A comparison of the surfaces of the as-received and laser-remelted coatings is shown in Fig. 3. The Al-dendrites of the as-received coating show a different dendritic pattern depending on the dendrite growth directions. Figure 3(a) shows the three-fold dendritic pattern of the as-received coatings with a spangle size of the order of 1 mm. The laser-remelted coating shows no visible spangle topography and is smooth with a lot of porosity defects on the surface (Fig. 3(d)). The secondary dendritic structure revealed at a higher magnification in the BE image mode is shown in Fig. 3(b,c). BE images of the laser-remelted coating (Fig. 3(e,f)) reveal a higher homogeneity in comparison to the as-received coating, where the secondary dendrite structure is much finer. The secondary dendrite-arm spacing for commercial coatings is typically in the range 6–10 µm, while on laser-remelted coating it is 0.5–1.0 µm.

**Figure 4:**
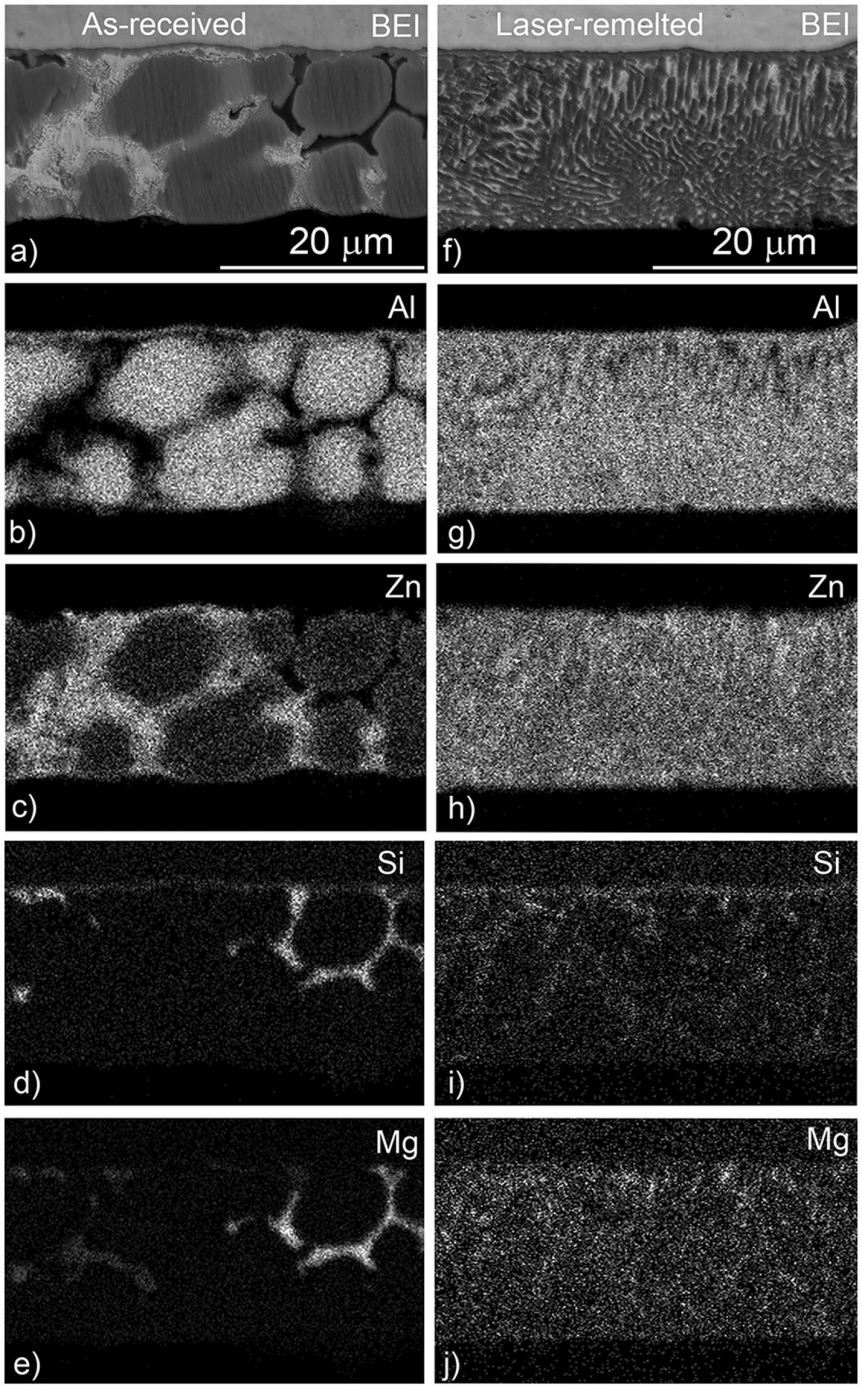
SEM/EDS X-ray mapping of Zn-55%Al-2%Mg-1.5%Si as-received (**a**–**e**) and laser remelted (**f**–**j**) coatings.

SEM/EDS analyses showing the distribution of the elements in the coating cross-sections are provided in Fig. 4. Segregation effects typical of commercial coatings, including Al-rich dendrites and Zn-Mg-Si-rich interdendritic regions, are very obvious for the as-received coating. On the other hand, these segregation effects are no longer evident on the laser-remelted coating. There is some fine-scale partitioning evident, and some localized concentrations of Si and Mg, most likely associated with the Si and Mg_2_Si phases. However, in the remelted coating the dendrite structure has been replaced by a much finer and more homogenous structure. There still appears to be a dendritic structure, but the dendrite-arm spacing in this case is much smaller, of the order of 1 µm, as compared to 10 µm in the as-received coating. The EDS X-ray maps demonstrate a more uniform distribution of phases throughout the coating.

### EBSD analysis

**Figure 5:**
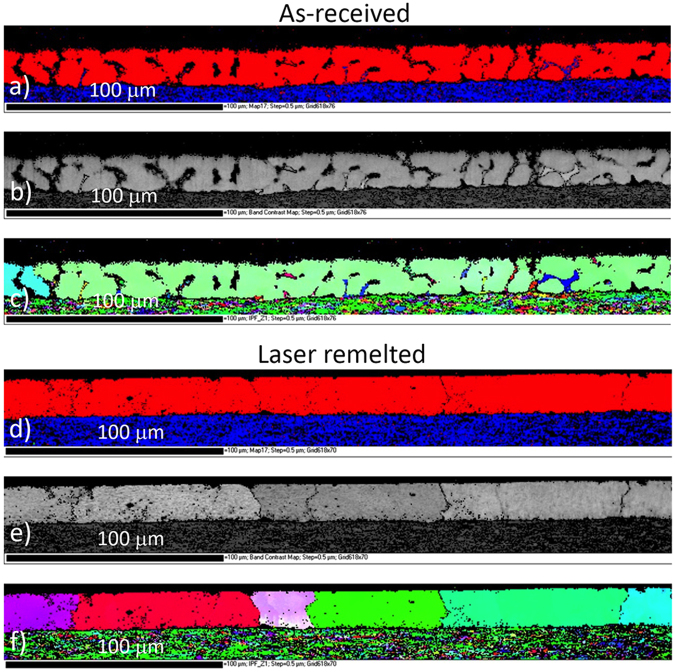
Cross-section EBSD analysis of as-received coatings and laser-remelted, (**a**) and (**d**) phase map, (**b**) and (**e**) band contrast and (**c**) and (**f**) IPF.

The EBSD results show the dendrite structure of the as-received coating with large grains in the mm range (Fig. 5). The as-received surface of the coatings is uneven due to the solidification shrinkage between the dendrites. Primarily, the dendrites are rich in Al, while the MgSi_2_ phase nucleates on the intermetallic layer in the interdendritic channels. The remaining liquid becomes progressively enriched in Mg and Zn until a complex mixture of binary Al-Zn eutectic and ternary Zn-MgZn_2_-Al eutectics eventually form in the interdendritic spaces. In the case of the laser-remelting coating, the rapid remelting and quenching causes grain refinement, with the coarse structure being replaced by smaller grains with a much finer dendrite structure. The sizes of the grains are 50–200 µm.

**Figure 6:**
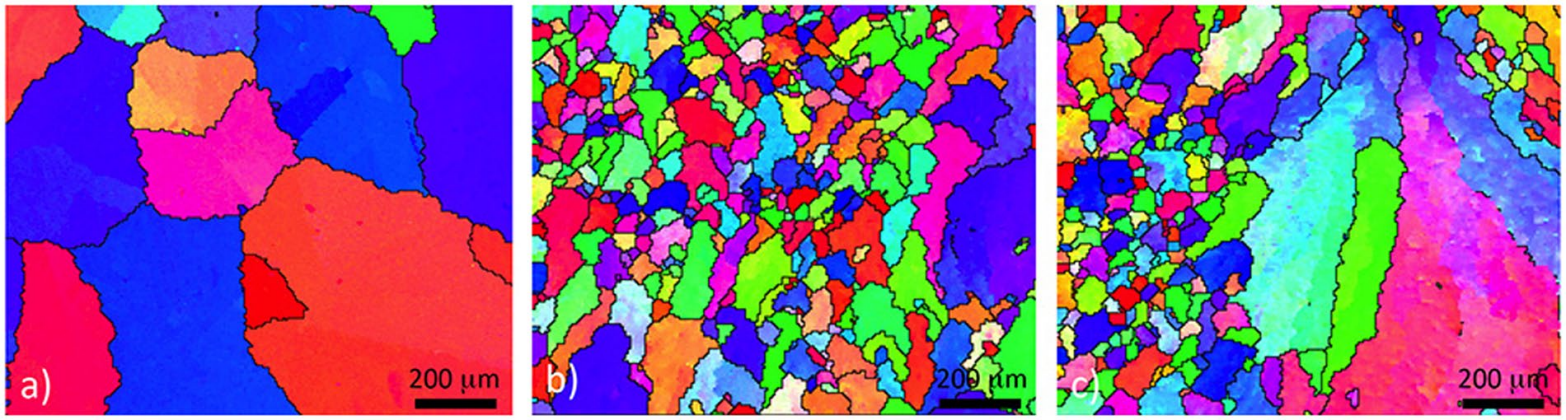
Top-view EBSD mapping IPF in Z direction; (**a**) as-received and (**b**) and (**c**) laser-remelted.

A top-view EBSD analysis is shown in Fig. 6. In the as-received coatings, no preferred texture was identified and the same applies to the laser-treated coating. However, there is clear evidence of a reduction in the size of the primary Al-dendrites (spangle size) following the laser remelting. The as-received coating shows a variation in the orientation along a dendrite arm. This is shown by gradual changes of the colour code in Fig. 6(a). The misorientation value from the centre of the dendrites to the grain boundary is usually in the range of 1 to 2 degrees, sometimes even 5 degrees. The misorientation phenomenon is known in the literature and is explained by the thermal stresses induced by differential thermal expansion of the coatings and the steel substrate or micro-segregation during solidification of the coating^5,6^.

Typically, the laser-remelted coating has much smaller primary Al-dendrites (see Fig. 6(b)). There are some large grains occurring during rapid solidification, as shown in Fig. 6(c), and the misorientation associated with large grains is significant and sometimes exceeds 10 degrees. These phenomena might be related to non-uniform heat transfer during cooling or due to the local enrichment of certain elements.

**Figure 7:**
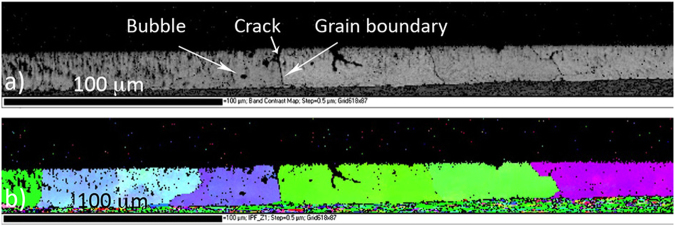
Cross-section EBSD analysis of laser-remelted coating, (**a**) band contrast, (**b**) IPF.

Some cracking and porosity are evident in the laser-remelted coating (Fig. 3(e,f)). The EBSD results show that the cracks typically occurred at the grain boundaries of the newly formed grains (see Fig. 7). Zn is present in the interdendritic regions of the coating microstructure, both as MgZn_2_ phase and also as relatively pure Zn. Zn has a high vapour pressure, and so it is possible that some Zn may form as vapour in the coating during melting, particularly where there is a cavity present. However, given that the peak temperature of the melted coating does not exceed 650 °C, and the coating is in a molten state for very short period, it is unlikely that Zn vapour formation and expansion is the primary cause of porosity. Porosity most likely occurring due to the fact that some small cavities are already present in between the dendrites and during the laser treatment these cavities transform into gas bubbles. There is some evidence that the porosity boundaries are enriched in Si. This is not surprising as the Mg_2_Si phase forms in the interdendritic regions and this is where the cavities are likely to form on solidification. These defects, and particularly the association of cracks with grain boundaries, suggest that there may be a detrimental effect on the coating’s ductility.

Ignoring the surface porosity and larger cracks, the laser treated coatings typically have a smoother surface (lower surface roughness) than the as-received material. This is because the relatively large scale aluminium dendrites are melted and replaced with a much finer scale aluminium dendrite network.

### Coating’s Ductility on Bending

**Figure 8:**
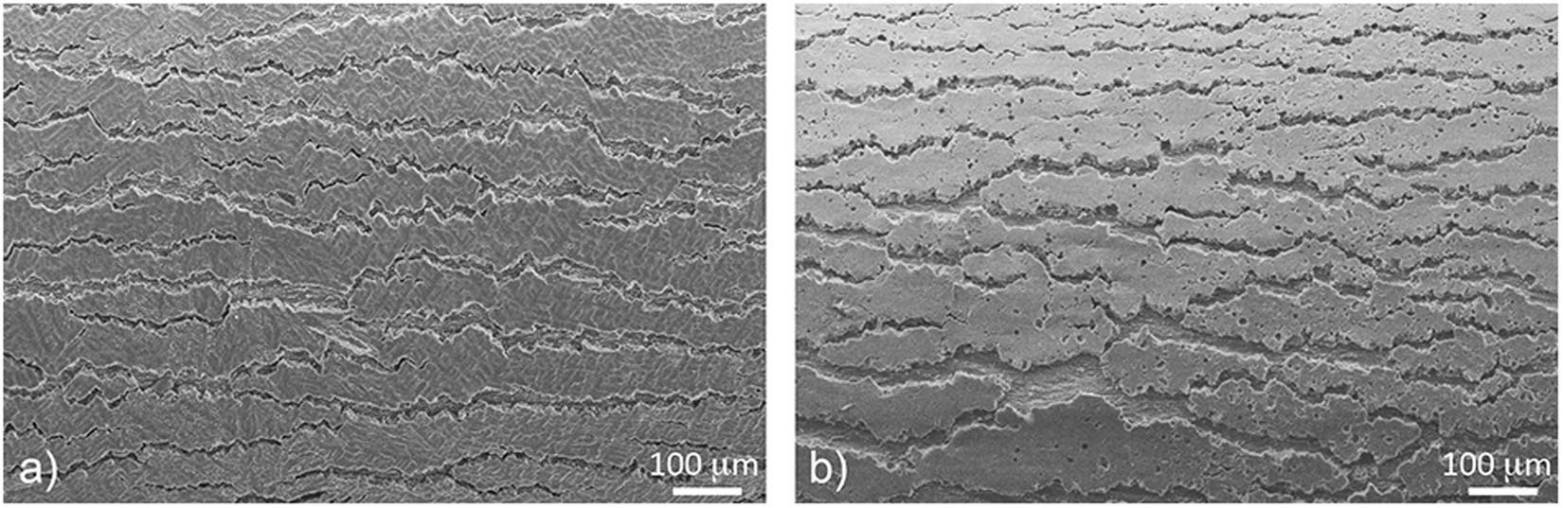
Appearance of crack for (**a**) as-received coating and (**b**) laser-remelted coating after 2T bend testing.

The coating’s ductility was evaluated using the 2T bend test, where coated samples are formed around a mandrill with a radius equivalent to the thickness of the steel substrate (in this case, 0.42 mm). Figure 8 shows SE images of the formed coating surfaces. The as-received coating shows typical cracks running parallel to the bend axis. These cracks are extensive, with edges showing some association with the dendritic facets. In the case of the laser-remelted coating, the extent of the cracking is not dramatically worse, with a similar crack area, crack morphology and direction evident. We measured the exposed steel (crack area) on several images (8.8 mm^2^) and found out that 3.8% and 4.6% of steel area was exposed at as-received and laser-remelted samples, respectively. Furthermore, laser-remelted coating doesn’t show any deterioration in terms of coating de-bonding from the substrate. Apparently, the extensive porosity and intergranular cracking does not lead to catastrophic consequences in terms of the coating’s ductility. Cracks in such coatings and at the bend radius used typically form in both coating overlay and intermetallic alloy layer, such that the substrate steel is exposed to environment in numerous locations. This is why cracking at bends is an important factor. Exposed steel accelerates the corrosion of the coating, and leads to rust staining at bends.

## Conclusions

The findings of the investigation can be summarised in the following conclusions:

- Laser remelting of the Al-Zn-Mg-Si coating and rapid self-quenching from the steel substrate produces a finer grain size, and a microstructure that is substantially refined and homogenized with respect to the phase distribution. Future work will investigate whether this refinement of the microstructure and homogenisation of the phase distribution will result in an improved corrosion performance.
- EBSD cross-section maps reveal cracks that occur on the newly formed grain boundaries. Also, some porosity arising from the gas bubbles trapped in the layer were found. All the structural defects have a negative effect on the coating properties and need to be avoided.
- EBSD top-view maps show no preferred orientation in the as-received layer, nor in the laser-remelted layer. The size of the grains in the laser-remelted layer is typically 10 times smaller than in the as-received layer. However, some large grains can also appear in the microstructure. Grain orientation, on the other hand, has no correlation with the previous non-refined grains.
- As-received and laser-remelted layers have some misorientation inside the dendrites. Some large grains of the laser-treated layer have an even larger misorientation along the growth direction.
- Despite the presence of significant porosity and intergranular cracking in the laser-treated coating, it does not result in significantly worse bend-ductility performance, as measured by the 2T bend test.

While the laser-remelted coatings are characterized by some porosity and cracking defects, this new technology offers the promise of a highly refined microstructure with the potential for improved corrosion performance.
